# DetectIS: a pipeline to rapidly detect exogenous DNA integration sites using DNA or RNA paired-end sequencing data

**DOI:** 10.1093/bioinformatics/btab366

**Published:** 2021-05-12

**Authors:** Luigi Grassi, Claire Harris, Jie Zhu, Colin Hardman, Diane Hatton

**Affiliations:** Biopharmaceutical Development, BioPharmaceuticals R&D, AstraZeneca, Cambridge, CB21 6GH, UK; Biopharmaceutical Development, BioPharmaceuticals R&D, AstraZeneca, Cambridge, CB21 6GH, UK; Biopharmaceutical Development, BioPharmaceuticals R&D, AstraZeneca, Gaithersburg, MD 20878, USA; Data Science & Artificial Intelligence, BioPharmaceuticals R&D, AstraZeneca, Cambridge, CB21 6GH, UK; Biopharmaceutical Development, BioPharmaceuticals R&D, AstraZeneca, Cambridge, CB21 6GH, UK

## Abstract

**Motivation:**

Recombinant DNA technology is widely used for different applications in biology, medicine and bio-technology. Viral transduction and plasmid transfection are among the most frequently used techniques to generate recombinant cell lines. Many of these methods result in the random integration of the plasmid into the host genome. Rapid identification of the integration sites is highly desirable in order to characterize these engineered cell lines.

**Results:**

We developed detectIS: a pipeline specifically designed to identify genomic integration sites of exogenous DNA, either a plasmid containing one or more transgenes or a virus. The pipeline is based on a Nextflow workflow combined with a Singularity image containing all the necessary software, ensuring high reproducibility and scalability of the analysis. We tested it on simulated datasets and RNA-seq data from a human sample infected with Hepatitis B virus. Comparisons with other state of the art tools show that our method can identify the integration site in different recombinant cell lines, with accurate results, lower computational demand and shorter execution times.

**Availability and implementation:**

The Nextflow workflow, the Singularity image and a test dataset are available at https://github.com/AstraZeneca/detectIS.

**Supplementary information:**

Supplementary data are available at *Bioinformatics* online.

## 1 Introduction

Recombinant DNA technology can be used to generate transgenic animals, plants and cell lines, widely used for different applications in biology, medicine and biotechnology (Ghaderi *et al.*, 2012; Khan *et al.*, 2016). Therapeutic proteins with complex post-translational modifications are normally expressed in mammalian cell lines (Walsh, 2018; Zhu and Hatton, 2018). Viral transduction and plasmid transfection are methods largely used to establish recombinant cell lines (Kim and Eberwine, 2010; Lee *et al*., 2018) and typically result in random integration of the transgene construct into the host genome. The identification of the transgene integration site (IS) is important for the characterization of stable recombinant cell lines and, can reveal regulatory features relevant for transgene expression. It can also detect aberrant transgene–host fusion proteins, potentially caused by the plasmid integrating in the proximity of protein-coding genes. Understanding ISs can identify integration ‘hot spots’, i.e. genomic sites conferring high expression of the transgene and data from multiple experiments can be used for the design of targeted ISs. Moreover, as the transgene ISs are unique for an individual transfection event, the IS information can be used to design PCR experiments to assess the clonality of a cell line (Sommeregger et al., 2013). Inverse PCR (Liang et al., 2008; Uemura et al., 2014), splinkerette-PCR (Uren et al., 2009) and targeted locus amplification (de Vree *et al.*, 2014) are techniques specifically designed to localize ISs in host genomes. High-throughput sequencing (HTS) experiments have been successfully used to localize a similar biological event: the viral ISs in host genomes (Chen *et al.*, 2019). Moreover, several studies have proved the usefulness of HTS in localizing plasmid ISs in stable cell lines (Brett *et al.*, 2011; Lambirth et al., 2015; Srivastava et al., 2014). Although pipelines have been developed for detecting viral integration sites, some of them are specifically designed for the human genome reference sequence. Moreover, all the tools require the preparation of indexes specific for each host and exogenous DNA element.

We present detectIS, a pipeline to detect the ISs in paired end (PE) HTS experiments (either DNA or RNA sequencing data). It can be directly used with different host and exogenous DNA references, without the need of creating a specific index. Consequently, it is suitable for different applications, for example detecting ISs of plasmids in stable cell lines, either clones or pools, as well as locating viruses integrated in any host genome. The speed of execution makes the detectIS pipeline well-suited for quickly screening HTS data from panels of different cell lines generated during the cell line development process for therapeutic protein manufacture, enabling the detection of cell lines with undesirable transgene fusion sequences.

## 2 Materials and methods

DetectIS (Supplementary Fig. S1) consists of three main steps. PE reads are aligned, in single-end mode onto the exogenous sequence reference (i.e. transgene, plasmid or viral sequences). Reads with any overlap with the exogenous reference sequence are subsequently aligned, in single-end mode, to the host genome reference. The alignment is made by using the Minimap2 program (Li, 2018). Finally, a *Perl* script integrates the four alignment results looking for potential ISs. ISs can be identified by split reads—read pairs in which at least one read has a part mapping to the host genome and the remaining part mapping to the plasmid/transgene, and chimeric reads, read pairs in which one of the two reads is mapped to the host genome and the other one to the plasmid/transgene. The pseudocode of the subroutines used by the *Perl* script is reported in Supplementary Figures S2–S9. Final results are provided as a txt file detailing all the potential ISs and the number of supporting split and chimeric read pairs. The same information is also reported in a markdown file that can be converted to a pdf and/or html file. All the steps of the detectIS pipeline are embedded in a Nextflow (Di Tommaso *et al.*, 2017) workflow that, together with the Singularity (Kurtzer *et al.*, 2017) container ensures reproducibility and scalability from a single PC/workstation to high-performance computational (HPC) environments.

## 3 Usage

In order to use the workflow, the user has to create a configuration file specifying the reference host genome and exogenous sequence references, the directory containing the raw data and the output directory. The analysis can be executed locally or in an HPC environment, in the latter scenario the user also has to specify the cluster executor. A configuration file is provided to analyze a test dataset and can be used as a template for other analyses.

The recipe of the Singularity image with all the necessary software is also supplied. A bash script is also given to analyze a test dataset without Nextflow and can be used as a template for analysis in local environments.

### 3.1 Comparison with existing tools for structural variant identification

In order to test the functionality of detectIS and the accuracy of its results, we simulated random integrations of a plasmid in a Chinese hamster ovary (CHO) scaffold, exploring different modalities of transgene size, depth of sequencing coverage and read length. We compared the results of detectIS with the ones derived by other tools for viral detection, that are able to use host references different from human. SeekSV (Liang et al., 2017) is a program designed to identify ISs and other structural variants in RNA-seq and DNA-seq experiments and was one of the best performing tools for identifying viral integrations in a recent study (Chen *et al.*, 2019). BatVI (Tennakoon and Sung, 2017) is a sensitive and fast tool used for the detection of viral integrations that, similarly to detectIS, uses a subtractive strategy where raw reads are aligned to the viral reference genomes in the first instance, and the partially mapped reads are then aligned to the host reference genome to detect viral integrations. SurVirus (Rajaby et al., 2021) is a recently published repeat-aware virus integration caller. The detectIS results are among the ones with highest precision and sensitivity in most of the simulated experiments with sequenced read of lengths 250 and 150 bases (Supplementary Figs S10A–F, S11–AF, Supplementary Tables S1–S3). Minimap2 works with read length of 100 bases or higher (Li, 2018) and, for this reason, 100 bases is the lowest read length compatible with detectIS. In this simulated scenario, the tool is less precise and sensitive than SurVirus and SeekSV for sequence coverages of 5× and 10×, but performs similarly at higher coverage (Supplementary Figs S10–GI, S11G–I, Tables Supplementary S1–S3). The execution times of the analyses are similar for detectIS, SurVirus and BatVI and higher for SeekSV in all the simulated experiments (Supplementary Fig. S12). DetectIS has the lowest computational demands with the lowest CPU times in all the simulated experiments (Supplementary Fig. S13). It is also notable that detectIS can be executed without the reference index generation, a time consuming step required by all the other tools (Supplementary Fig. S14). The integration sites detected by all the used tools have an average discrepancy of a few nucleotides in respect to the original sites (Supplementary Fig. S15). In the simulated integrations, plasmid and host had the same orientation 5′→3′ and this feature was captured by all the tools.

We extended the comparison to publicly available RNA-seq experiments of four hepatitis B virus (HBV) positive hepatocellular carcinoma cell lines with verified chimeric viral-human transcripts (Lau et al., 2014). In this analysis, SurVirus terminated with a segmentation fault error in all the four analyzed experiments and produced an empty final result file in three of them. Analogously, BatVI produced a final result file for only one of the four analyzed experiments, for this reason, we could compare only the results generated by detectIS and seekSV. We defined true positives as ISs that supported the chimeric viral-human transcripts verified in the study of Lau *et al.* (2014), with a tolerance of 50 nucleotides (Supplementary Table S4). The two tools gave similar results in term of precision, sensitivity (Supplementary Fig. S16A, Supplementary Table S5) and difference from the real data (Fig. S16B) with a significantly shorter running time for detectIS (Supplementary Fig. S16C and D). This difference in running times can be justified by the fact that the two pipelines are based on different programs and strategies, with seekSV looking for all potential structural variants while detectIS uses a subtractive strategy and is designed to specifically identify variants affecting the exogenous DNA (plasmid/virus). The results presented in this study demonstrate that detectIS is able to identify integration sites in HTS experiments, in a short time without high demands on computational resources. The benchmark analysis indicates that a longer read length improves detectIS precision and sensitivity in experiments made at a lower coverage. The usage of the Minimap2 program for the alignment gives the possibility of running the analysis without any index preparation step and makes the pipeline unique among all the existing programs for viral integration. Due to its versatility, detectIS can be executed to identify viral integration sites in transcriptome or genome sequencing experiments and identify the ISs of plasmids inserted into stable cell lines from HTS experiments routinely made to exclude the presence of variants in transgenic transcripts during clone selection (Harris *et al.*, 2019; Lin et al., 2019).

*Financial Support*: none declared.

*Conflict of Interest*: none declared.

## Supplementary Material

btab366_Supplementary_Data
