# Supplementary material for: DetectIS: a pipeline to rapidly detect exogenous DNA integration sites using DNA or RNA paired-end sequencing data
Source: Bioinformatics. 2021 May 12;37(22):4230–2. doi: 10.1093/bioinformatics/btab366 (PMC9502153; doi:10.1093/bioinformatics/btab366)
Supplement: btab366_Supplementary_Data [file btab366_supplementary_data.pdf]

DetectIS: a pipeline to rapidly detect exogenous DNA integration sites using DNA or RNA paired-end sequencing data

Luigi Grassi, Claire Harris, Jie Zhu, Colin Hardman and Diane Hatton

Supplementary materials

DetectIS pipeline and Perl script used to analyse the alignment results

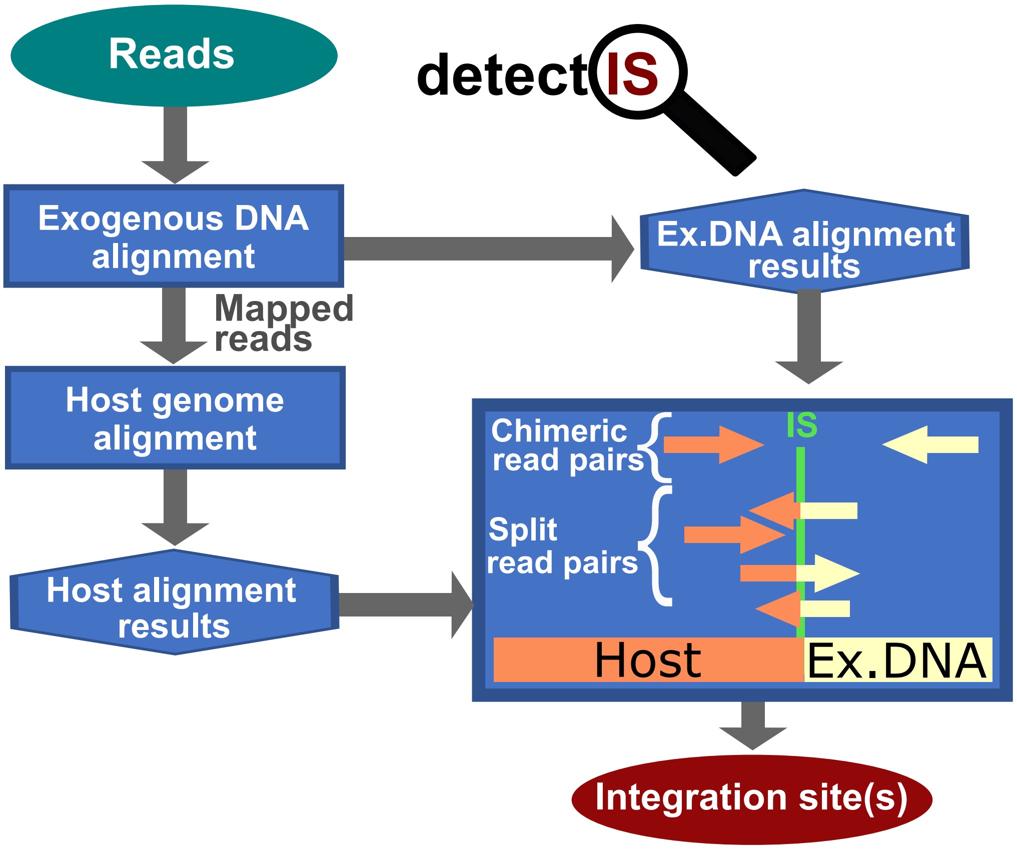

Figure S1 : DetectIS pipeline. Reads are aligned in single-end mode to the exogenous reference (plasmid/transgene); the ones overlapping the exogenous reference are then aligned to the host reference in single-end. The alignment results are finally analysed to identify chimeric and split read pairs.

```
detect_split_reads: identifies potential split reads in the host alignment results

verify_split_reads: identifies split reads in the exogenous DNA alignment results

merge_split_pairs: combines the result of read pairs

verify_spreads_is: classifies split read pairs

if (no integration site is identified by split reads) {

  detect_chimeric_reads: identifies potential chimeric reads in the host alignment results

  filter_chimeric_reads: identifies chimeric reads in the exogenous DNA alignment results

  count_and_collapse_chimeric_reads: groups chimeric reads
}
```

Figure S2 : Subroutines used by the detectIS.pl script to identify the integration sites in the alignment results on the host and exogenous references. The script looks for integration sites supported by split reads. Split and chimeric read pairs supporting the integration sites are then counted. If no integration site is identified by split reads the script looks for chimeric read pairs.

```
[Open host alignment paf file]

if (a read is:
  mapped only in one location AND
  the mapping length is shorter than the read length AND
  the mapping quality is higher than threshold)
{
  it is a potential split read
}

[Close host alignment paf file]
```

Figure S3 : Pseudocode of the subroutine `detect_split_reads`, used by the detectIS.pl script to identify potential split reads in the host alignment results.

```
[Open exogenous DNA alignment paf file]

if (a read is:
  identified as potential split read in the host alignment AND
  is mapped only in one location in the exougenous DNA AND
  the host-exogenous overlap/distance is shorter than threshold)
{
  Save the potential integration site and the host-exogenous DNA orientation
}

Count reads supporting each identified integration site

[Close exogenous DNA alignment paf file]
```

Figure S4 : Pseudocode of the subroutine `verify_split_reads`, used by the detectIS.pl script to identify potential split reads in the exogenous DNA alignment results.

```
[Combine the results of the read pairs]

[Filter out integration sites supported by a number of pairs below the threshold]
```

Figure S5 : Pseudocode of the subroutine `merge_split_pairs`, used by the detectIS.pl script to merge the single-end results to paired-end results.

```
[Remove split reads supporting different integration sites]

[Open all the paf files]

if (a read pair is made by 2 split reads) {
    it is classified as R1R2
}
else if  (the R1 read is a split read AND
          the R2 is mapped within 5 read lengths of the integration site) {
          it is classified as R1
}
else if (the R2 read is a split read AND
          the R1 is mapped within 5 read lengths of the integration site) {
          it is classified as R2
}
else if (only one read is split and the other does not support the integration site) {
        it is classified as R0
}

if (a read pair is made of a read mapped to host AND
    the other mapped to the exogenous reference AND
    both are located within 5 read lengths of the integration site) {
    it is classified as chimeric
}

[Close all the paf files]
```

Figure S6 : Pseudocode of the subroutine `verify_spreads_is`, used by the detectIS.pl script to classify split and chimeric read pairs supporting the identified integration sites.

```
[Open the host alignment paf files]

if (only one read of the pair is mapped to the reference AND
    at least 90 % residues are matching AND
    the residues matching are at least 99% of the alignment block length AND
    the mapping quality is equal to 60 AND
    the read is mapped only in one location) {

    it is classified as potential chimeric

}

[Close the host alignment paf files]
```

Figure S7 : Pseudocode of the subroutine `detect_chimeric_reads`, used by the detectIS.pl script to identify potential chimeric reads in the host alignment results.

```
[Open exogenous DNA alignment paf file]

if (only one read of the pair is mapped to the reference AND
    at least 90 % residues are matching AND
    the residues matching are at least 99% of the alignment block length AND
    the read is mapped only in one location AND
    it is identified as potential split read in the host alignment) {

    it is classified as  chimeric

}
[Close exogenous DNA alignment paf file]
```

Figure S8 : Pseudocode of the subroutine `filter_chimeric_reads`, used by the detectIS.pl script to identify chimeric reads in the exogenous DNA alignment results.

```
[Open host and exogenous DNA alignment paf files]

[Group chimeric read pairs in chromosomic intervals defined as 5 times the average read lenght]

[Close host and exogenous DNA alignment paf files]
```

Figure S9 : Pseudocode of the subroutine `count_and_collapse_chimeric_reads`, used by the detectIS.pl script to group chimeric reads in chromosome intervals.

Simulated random integration datasets

The random integration of the CelTag plasmid, downloaded from https://www.addgene.org/66562/sequences/#depositor-full, in the CHO scaffold_0 (1:224834208) downloaded from http://ftp.ensembl.org/pub/release-103/fasta/cricetulus_griseus_chok1gshd/dna/Cricetulus_griseus_chok1gshd.CHOK1GS_HDv1.dna.toplevel.fa.gz, was simulated in three different scenarios:

```
1) a random selection of the plasmid covering approximately half of its length,
0.5C sample (2842 nucleotides);

2) a random selection of the plasmid covering approximately its total length,
1C sample (5741 nucleotides);

3) a random selection of the plasmid covering approximately two lengths of the plasmid,
2C sample (11426 nucleotides).
```

Using the wgsim program (https://github.com/lh3/wgsim), for each scenario we simulated 3 replicates for 4 different coverage sequencing designs:

```
1)5X;
2)10X;
3)20X;
4)60X;
```

and 3 different read lengths:

```
1)100 nt;
2)150 nt;
3)250 nt.
```

In total we analysed 108 simulated datasets using 4 alternative tools: detectIS, seekSV, BatVI and SurVirus. The integration sites identified are considered true positives if they correspond to the coordinates of the original sequence used for the simulation, with a tolerance of 50 nucleotides. Integration sites identified at higher distance and/or in different chromosomes are considered false positives. Non reported integration sites of the original sequence used for the simulation are considered false negatives.

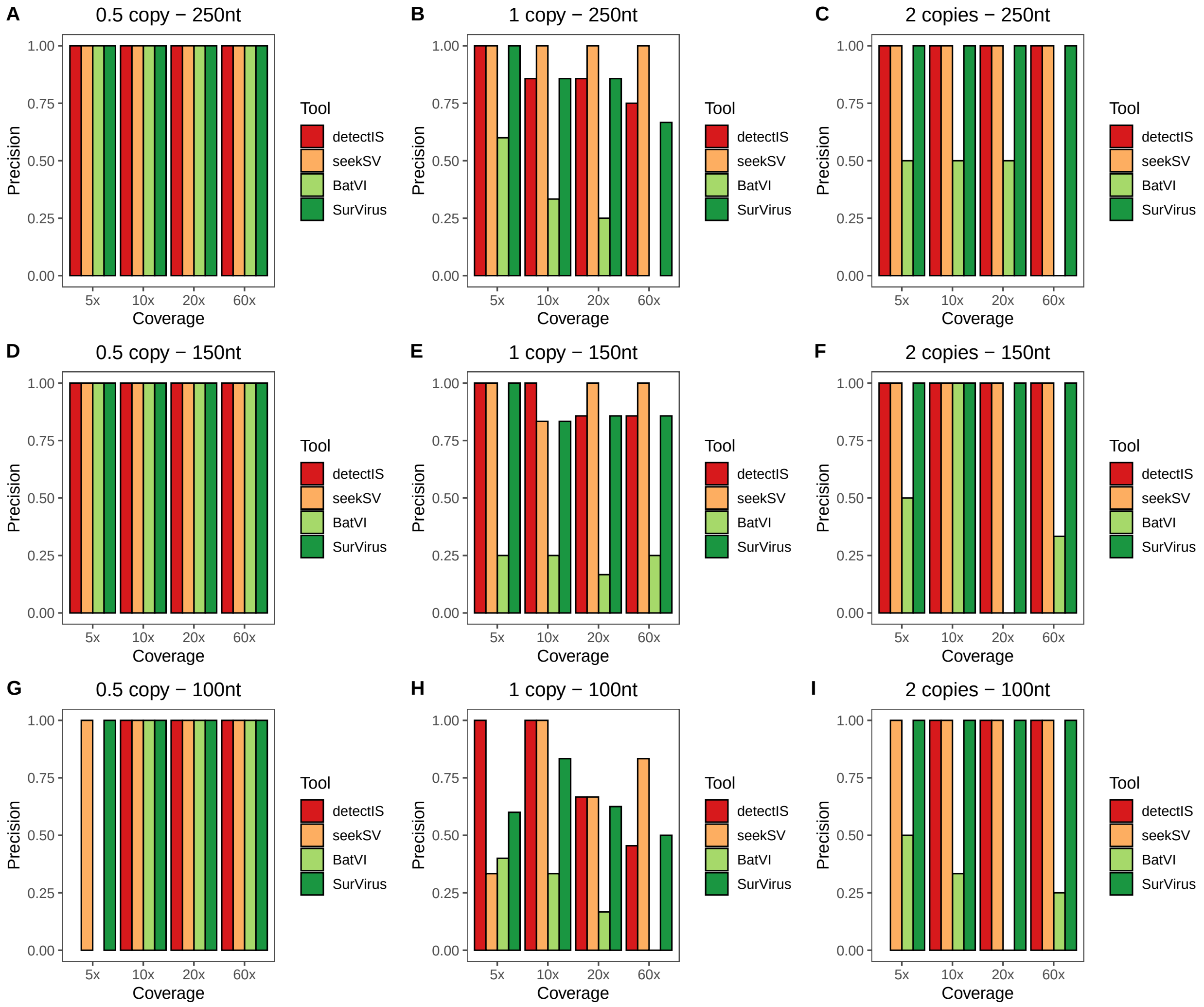

Figure S10 : Precision of the 4 tools, calculated as the number of true positives divided by the sum of true positives and false positives. For each coverage point we simulated three sequencing experiments with different insert sizes and different read lengths. A)0.5 vector copy with a read length of 250 nt; B)1 vector copy with a read length of 250 nt; C)2 vector copies with a read length of 250 nt; D)0.5 vector copy with a read length of 150 nt; E)1 vector copy with a read length of 150 nt; F)2 vector copies with a read length of 150 nt; G)0.5 vector copy with a read length of 100 nt; H)1 vector copy with a read length of 100 nt; I)2 vector copies with a read length of 100 nt.

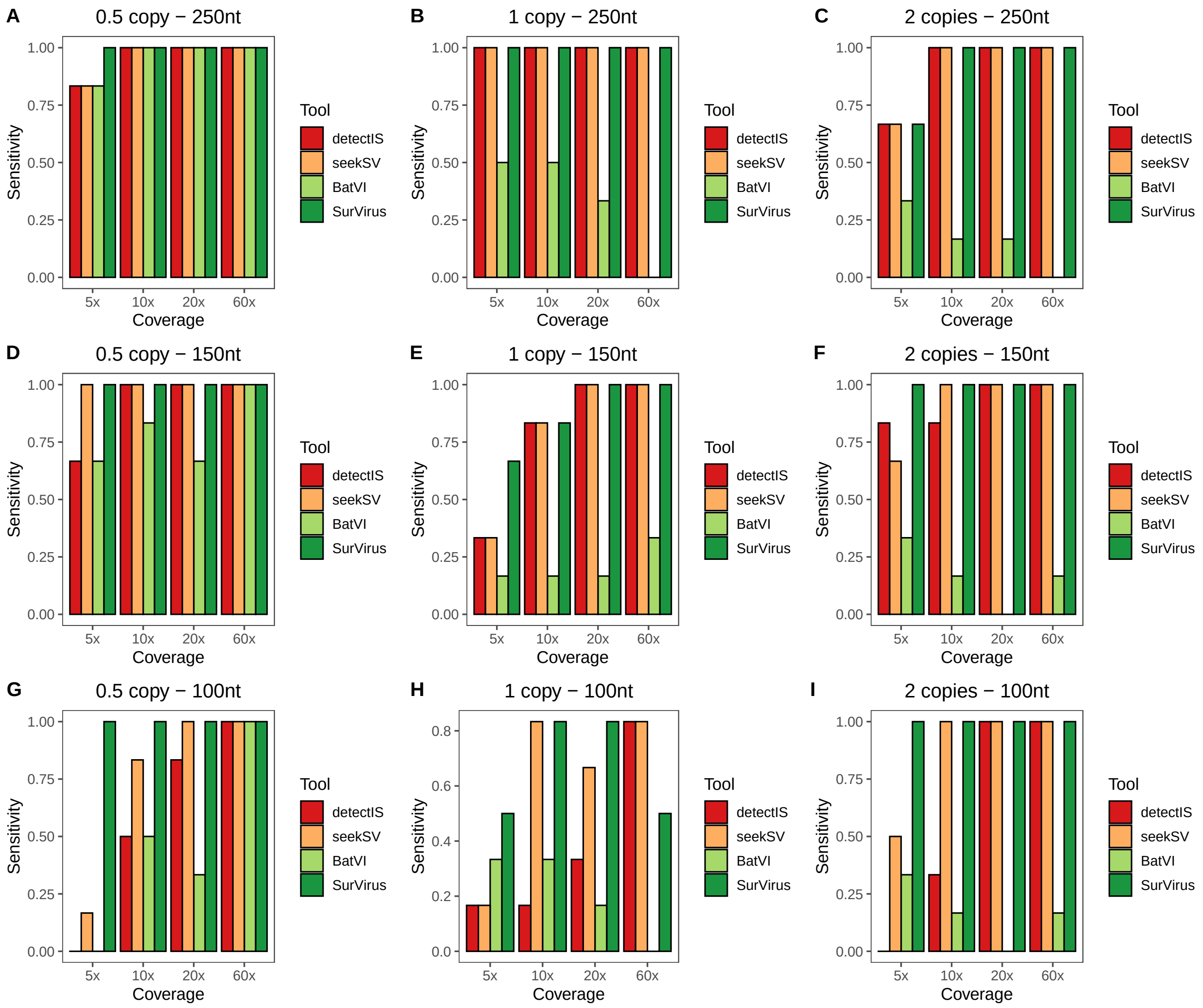

Figure S11 : Sensitivity of the 4 tools, calculated as the number of true positives divided by the sum of true positives and false negatives. For each coverage point we simulated three sequencing experiments with different insert sizes and different read lengths. A)0.5 vector copy with a read length of 250 nt; B)1 vector copy with a read length of 250 nt; C)2 vector copies with a read length of 250 nt; D)0.5 vector copy with a read length of 150 nt; E)1 vector copy with a read length of 150 nt; F)2 vector copies with a read length of 150 nt; G)0.5 vector copy with a read length of 100 nt; H)1 vector copy with a read length of 100 nt; I)2 vector copies with a read length of 100 nt.

Tool	Insert size	Read length	Sequencing coverage	True positives	False positives	False negatives
detectIS	0.5 copy	250 nt	5x	5	0	1
seekSV	0.5 copy	250 nt	5x	5	0	1
BatVI	0.5 copy	250 nt	5x	5	0	1
SurVirus	0.5 copy	250 nt	5x	6	0	0
detectIS	0.5 copy	250 nt	10x	6	0	0
seekSV	0.5 copy	250 nt	10x	6	0	0
BatVI	0.5 copy	250 nt	10x	6	0	0
SurVirus	0.5 copy	250 nt	10x	6	0	0
detectIS	0.5 copy	250 nt	20x	6	0	0
seekSV	0.5 copy	250 nt	20x	6	0	0
BatVI	0.5 copy	250 nt	20x	6	0	0
SurVirus	0.5 copy	250 nt	20x	6	0	0
detectIS	0.5 copy	250 nt	60x	6	0	0
seekSV	0.5 copy	250 nt	60x	6	0	0
BatVI	0.5 copy	250 nt	60x	6	0	0
SurVirus	0.5 copy	250 nt	60x	6	0	0
detectIS	0.5 copy	150 nt	5x	4	0	2
seekSV	0.5 copy	150 nt	5x	6	0	0
BatVI	0.5 copy	150 nt	5x	4	0	2
SurVirus	0.5 copy	150 nt	5x	6	0	0
detectIS	0.5 copy	150 nt	10x	6	0	0
seekSV	0.5 copy	150 nt	10x	6	0	0
BatVI	0.5 copy	150 nt	10x	5	0	1
SurVirus	0.5 copy	150 nt	10x	6	0	0
detectIS	0.5 copy	150 nt	20x	6	0	0
seekSV	0.5 copy	150 nt	20x	6	0	0
BatVI	0.5 copy	150 nt	20x	4	0	2
SurVirus	0.5 copy	150 nt	20x	6	0	0
detectIS	0.5 copy	150 nt	60x	6	0	0
seekSV	0.5 copy	150 nt	60x	6	0	0
BatVI	0.5 copy	150 nt	60x	6	0	0
SurVirus	0.5 copy	150 nt	60x	6	0	0
detectIS	0.5 copy	100 nt	5x	0	0	6
seekSV	0.5 copy	100 nt	5x	1	0	5
BatVI	0.5 copy	100 nt	5x	0	0	6
SurVirus	0.5 copy	100 nt	5x	6	0	0
detectIS	0.5 copy	100 nt	10x	3	0	3
seekSV	0.5 copy	100 nt	10x	5	0	1
BatVI	0.5 copy	100 nt	10x	3	0	3
SurVirus	0.5 copy	100 nt	10x	6	0	0
detectIS	0.5 copy	100 nt	20x	5	0	1
seekSV	0.5 copy	100 nt	20x	6	0	0
BatVI	0.5 copy	100 nt	20x	2	0	4
SurVirus	0.5 copy	100 nt	20x	6	0	0
detectIS	0.5 copy	100 nt	60x	6	0	0
seekSV	0.5 copy	100 nt	60x	6	0	0
BatVI	0.5 copy	100 nt	60x	6	0	0
SurVirus	0.5 copy	100 nt	60x	6	0	0

Table S1 : In the table are reported true positives, false positives and false negatives for the 0.5 vector copy simulated experiments, analysed using the four tools.

Tool	Insert size	Read length	Sequencing coverage	True positives	False positives	False negatives
detectIS	1 copy	250 nt	5x	6	0	0
seekSV	1 copy	250 nt	5x	6	0	0
BatVI	1 copy	250 nt	5x	3	2	3
SurVirus	1 copy	250 nt	5x	6	0	0
detectIS	1 copy	250 nt	10x	6	1	0
seekSV	1 copy	250 nt	10x	6	0	0
BatVI	1 copy	250 nt	10x	3	6	3
SurVirus	1 copy	250 nt	10x	6	1	0
detectIS	1 copy	250 nt	20x	6	1	0
seekSV	1 copy	250 nt	20x	6	0	0
BatVI	1 copy	250 nt	20x	2	6	4
SurVirus	1 copy	250 nt	20x	6	1	0
detectIS	1 copy	250 nt	60x	6	2	0
seekSV	1 copy	250 nt	60x	6	0	0
BatVI	1 copy	250 nt	60x	0	0	6
SurVirus	1 copy	250 nt	60x	6	3	0
detectIS	1 copy	150 nt	5x	2	0	4
seekSV	1 copy	150 nt	5x	2	0	4
BatVI	1 copy	150 nt	5x	1	3	5
SurVirus	1 copy	150 nt	5x	4	0	2
detectIS	1 copy	150 nt	10x	5	0	1
seekSV	1 copy	150 nt	10x	5	1	1
BatVI	1 copy	150 nt	10x	1	3	5
SurVirus	1 copy	150 nt	10x	5	1	1
detectIS	1 copy	150 nt	20x	6	1	0
seekSV	1 copy	150 nt	20x	6	0	0
BatVI	1 copy	150 nt	20x	1	5	5
SurVirus	1 copy	150 nt	20x	6	1	0
detectIS	1 copy	150 nt	60x	6	1	0
seekSV	1 copy	150 nt	60x	6	0	0
BatVI	1 copy	150 nt	60x	2	6	4
SurVirus	1 copy	150 nt	60x	6	1	0
detectIS	1 copy	100 nt	5x	1	0	5
seekSV	1 copy	100 nt	5x	1	2	5
BatVI	1 copy	100 nt	5x	2	3	4
SurVirus	1 copy	100 nt	5x	3	2	3
detectIS	1 copy	100 nt	10x	1	0	5
seekSV	1 copy	100 nt	10x	5	0	1
BatVI	1 copy	100 nt	10x	2	4	4
SurVirus	1 copy	100 nt	10x	5	1	1
detectIS	1 copy	100 nt	20x	2	1	4
seekSV	1 copy	100 nt	20x	4	2	2
BatVI	1 copy	100 nt	20x	1	5	5
SurVirus	1 copy	100 nt	20x	5	3	1
detectIS	1 copy	100 nt	60x	5	6	1
seekSV	1 copy	100 nt	60x	5	1	1
BatVI	1 copy	100 nt	60x	0	3	6
SurVirus	1 copy	100 nt	60x	3	3	3

Table S2 : In the table are reported true positives, false positives and false negatives for the 1 vector copy simulated experiments, analysed using the four tools.

Tool	Insert size	Read length	Sequencing coverage	True positives	False positives	False negatives
detectIS	2 copies	250 nt	5x	4	0	2
seekSV	2 copies	250 nt	5x	4	0	2
BatVI	2 copies	250 nt	5x	2	2	4
SurVirus	2 copies	250 nt	5x	4	0	2
detectIS	2 copies	250 nt	10x	6	0	0
seekSV	2 copies	250 nt	10x	6	0	0
BatVI	2 copies	250 nt	10x	1	1	5
SurVirus	2 copies	250 nt	10x	6	0	0
detectIS	2 copies	250 nt	20x	6	0	0
seekSV	2 copies	250 nt	20x	6	0	0
BatVI	2 copies	250 nt	20x	1	1	5
SurVirus	2 copies	250 nt	20x	6	0	0
detectIS	2 copies	250 nt	60x	6	0	0
seekSV	2 copies	250 nt	60x	6	0	0
BatVI	2 copies	250 nt	60x	0	4	6
SurVirus	2 copies	250 nt	60x	6	0	0
detectIS	2 copies	150 nt	5x	5	0	1
seekSV	2 copies	150 nt	5x	4	0	2
BatVI	2 copies	150 nt	5x	2	2	4
SurVirus	2 copies	150 nt	5x	6	0	0
detectIS	2 copies	150 nt	10x	5	0	1
seekSV	2 copies	150 nt	10x	6	0	0
BatVI	2 copies	150 nt	10x	1	0	5
SurVirus	2 copies	150 nt	10x	6	0	0
detectIS	2 copies	150 nt	20x	6	0	0
seekSV	2 copies	150 nt	20x	6	0	0
BatVI	2 copies	150 nt	20x	0	1	6
SurVirus	2 copies	150 nt	20x	6	0	0
detectIS	2 copies	150 nt	60x	6	0	0
seekSV	2 copies	150 nt	60x	6	0	0
BatVI	2 copies	150 nt	60x	1	2	5
SurVirus	2 copies	150 nt	60x	6	0	0
detectIS	2 copies	100 nt	5x	0	0	6
seekSV	2 copies	100 nt	5x	3	0	3
BatVI	2 copies	100 nt	5x	2	2	4
SurVirus	2 copies	100 nt	5x	6	0	0
detectIS	2 copies	100 nt	10x	2	0	4
seekSV	2 copies	100 nt	10x	6	0	0
BatVI	2 copies	100 nt	10x	1	2	5
SurVirus	2 copies	100 nt	10x	6	0	0
detectIS	2 copies	100 nt	20x	6	0	0
seekSV	2 copies	100 nt	20x	6	0	0
BatVI	2 copies	100 nt	20x	0	3	6
SurVirus	2 copies	100 nt	20x	6	0	0
detectIS	2 copies	100 nt	60x	6	0	0
seekSV	2 copies	100 nt	60x	6	0	0
BatVI	2 copies	100 nt	60x	1	3	5
SurVirus	2 copies	100 nt	60x	6	0	0

Table S3 : In the table are reported true positives, false positives and false negatives for the 2 vector copies simulated experiments, analysed using the four tools.

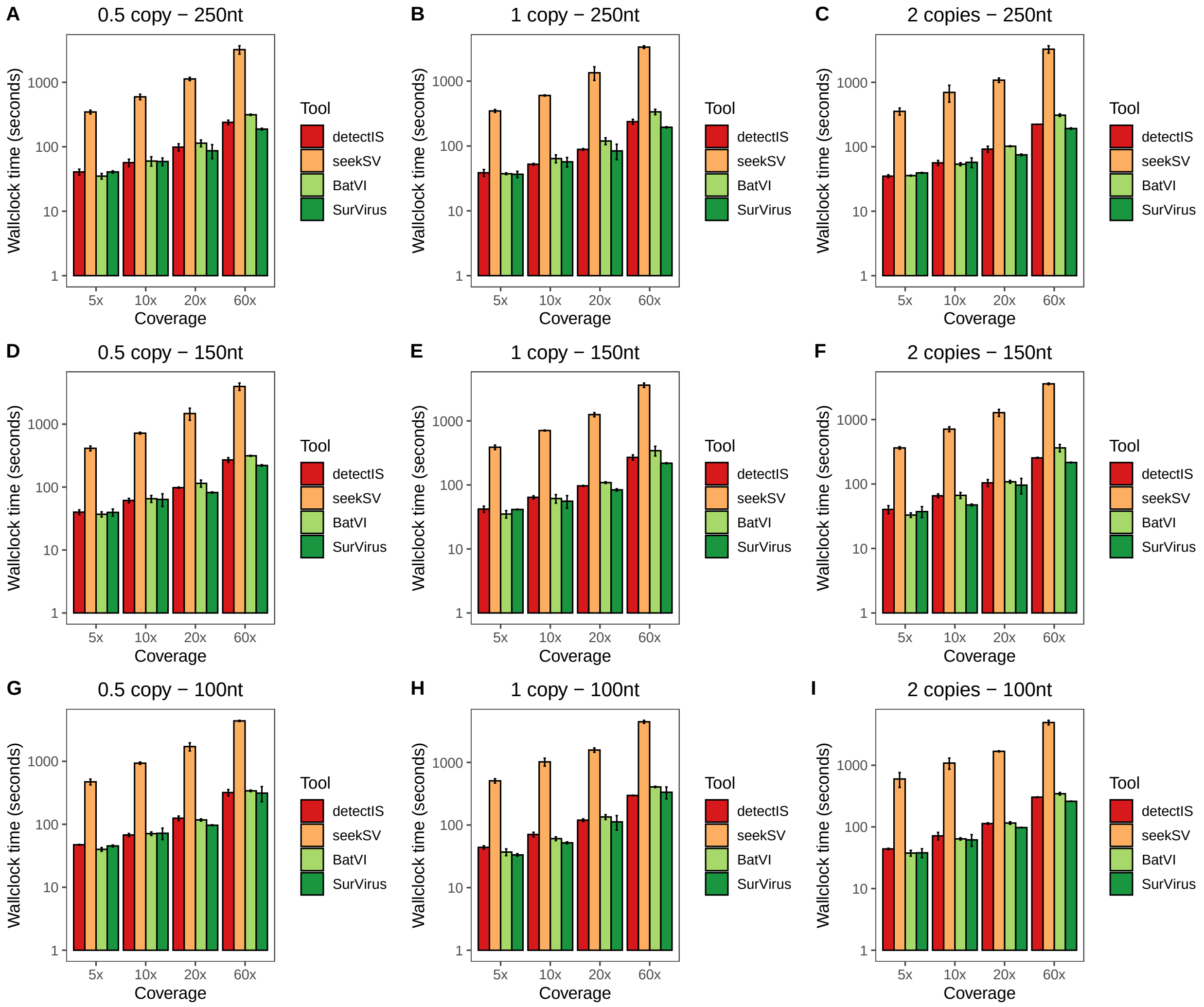

Figure S12 : Wall clock times derived using the qacct program. For each coverage point we simulated three sequencing experiments with different insert sizes and different read lengths. A)0.5 vector copy with a read length of 250 nt; B)1 vector copy with a read length of 250 nt; C)2 vector copies with a read length of 250 nt; D)0.5 vector copy with a read length of 150 nt; E)1 vector copy with a read length of 150 nt; F)2 vector copies with a read length of 150 nt; G)0.5 vector copy with a read length of 100 nt; H)1 vector copy with a read length of 100 nt; I)2 vector copies with a read length of 100 nt.

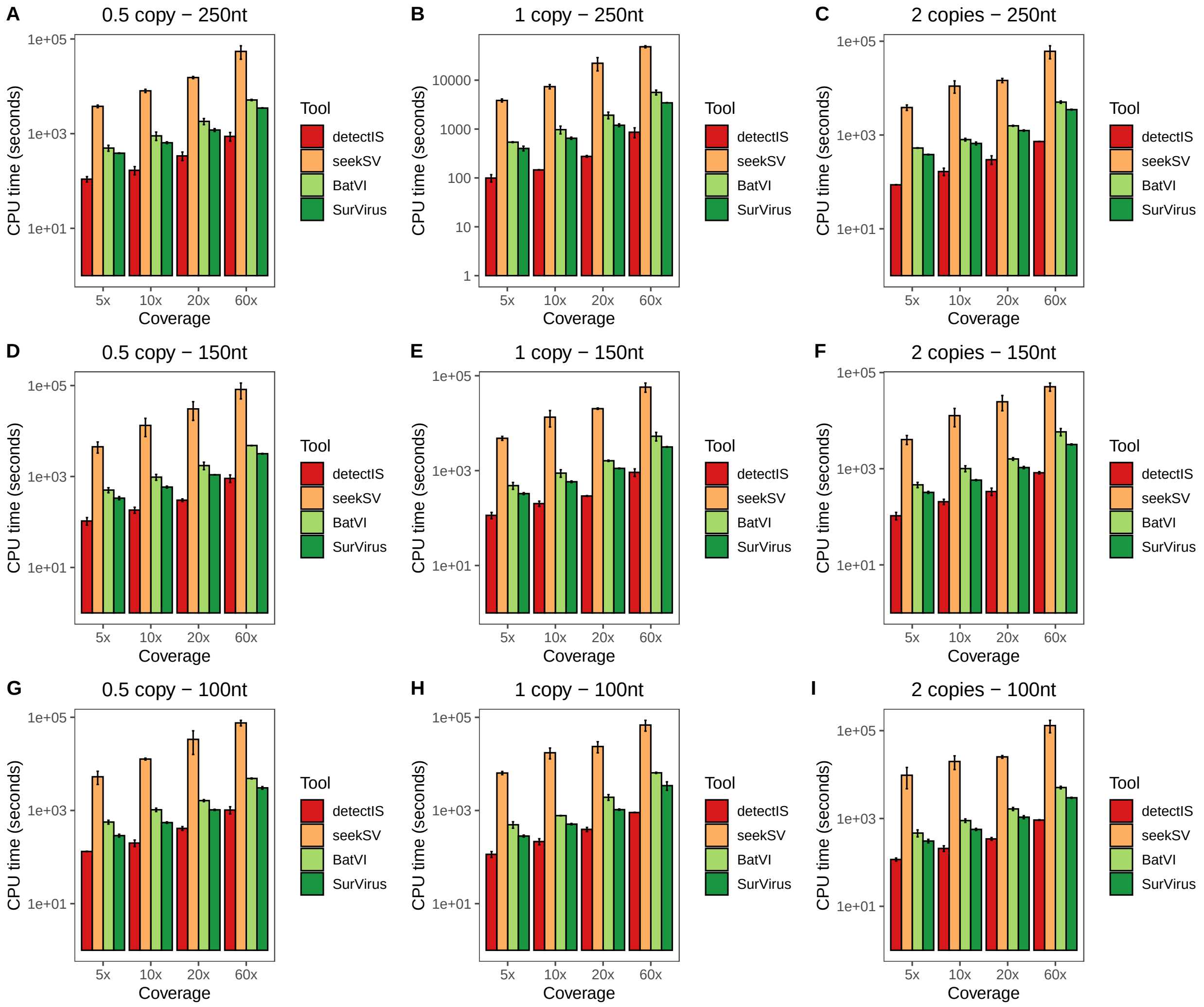

Figure S13 : CPU time of the different analyses, derived using the qacct program. For each coverage point we simulated three sequencing experiments with different insert sizes and different read lengths. A)0.5 vector copy with a read length of 250 nt; B)1 vector copy with a read length of 250 nt; C)2 vector copies with a read length of 250 nt; D)0.5 vector copy with a read length of 150 nt; E)1 vector copy with a read length of 150 nt; F)2 vector copies with a read length of 150 nt; G)0.5 vector copy with a read length of 100 nt; H)1 vector copy with a read length of 100 nt; I)2 vector copies with a read length of 100 nt.

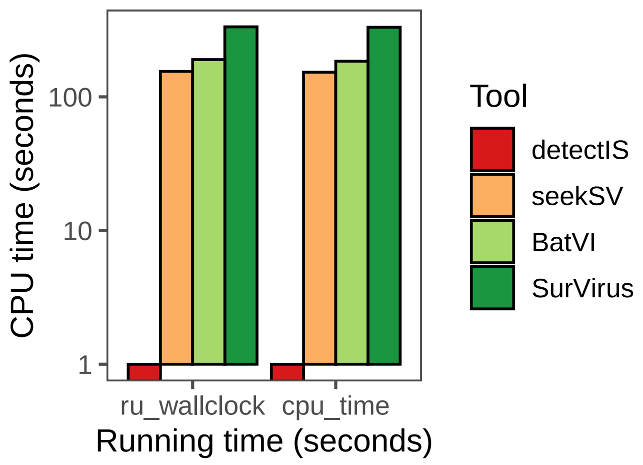

Figure S14 : Wall clock and CPU times of the index generation step, derived using the qacct program.

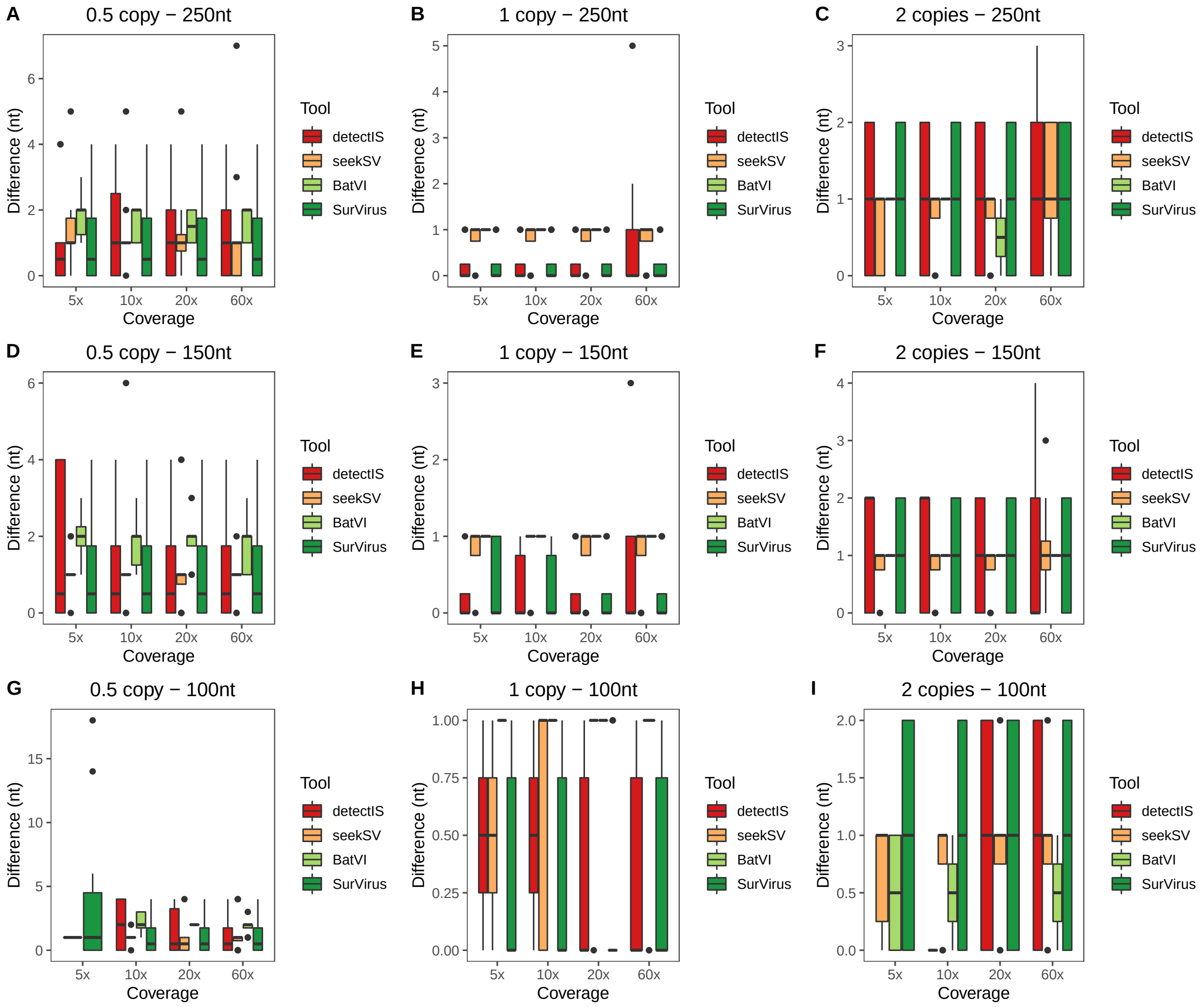

Figure S15 : Box plot reporting the absolute values of the differences in nucleotides of the true positives results respect to the real coordinates. For each coverage point we simulated three sequencing experiments with different insert sizes and different read lengths. A)0.5 vector copy with a read length of 250 nt; B)1 vector copy with a read length of 250 nt; C)2 vector copies with a read length of 250 nt; D)0.5 vector copy with a read length of 150 nt; E)1 vector copy with a read length of 150 nt; F)2 vector copies with a read length of 150 nt; G)0.5 vector copy with a read length of 100 nt; H)1 vector copy with a read length of 100 nt; I)2 vector copies with a read length of 100 nt.

RNA-seq experiments of Hepatitis B virus (HBV) positive Hepatocellular carcinoma cell lines.

Cell	HBV_coord	HBV_strand	Human_Chr	Human_Coord	Human_strand	Orientation
HKCI-4	467-711	+	chr4	54042613-54042654	+	HBV-Human
HKCI-4	94-285	+	chr5	1295686-1295717	+	Human-HBV
HKCI-4	1539-1622	+	chr8	41636407-41636543	+	HBV-Human
HKCI-4	241-459	+	chr13	114304790-114304969	+	HBV-Human
HKCI-4	1931-2190	+	chr9	126972125-126972204	-	Human-HBV
HKCI-5B	1212-1983	+	chr7	98934504-98934693	+	HBV-Human
HKCI-5B	1835-2021	+	chr16	30396945-30397035	+	Human-HBV
HKCI-7	1671-1811	+	chr4	99028393-99028423	-	HBV-Human
HKCI-7	1842-1936	+	chr11	69120112-69120143	-	Human-HBV
HKCI-7	1095-1164	+	chr11	69085793-69085826	-	HBV-Human
HKCI-11	1805-1658	-	chr4	53063790-53063967	+	Human-HBV

Table S4 : Sanger validated chimeric transcripts derived from the study http://dx.doi.org/10.1016/j.ccr.2014.01.030. The original human coordinates, downloaded from https://ars.els-cdn.com/content/image/1-s2.0-S1535610814000440-mmc2.xlsx have been converted from hg19 to hg38.

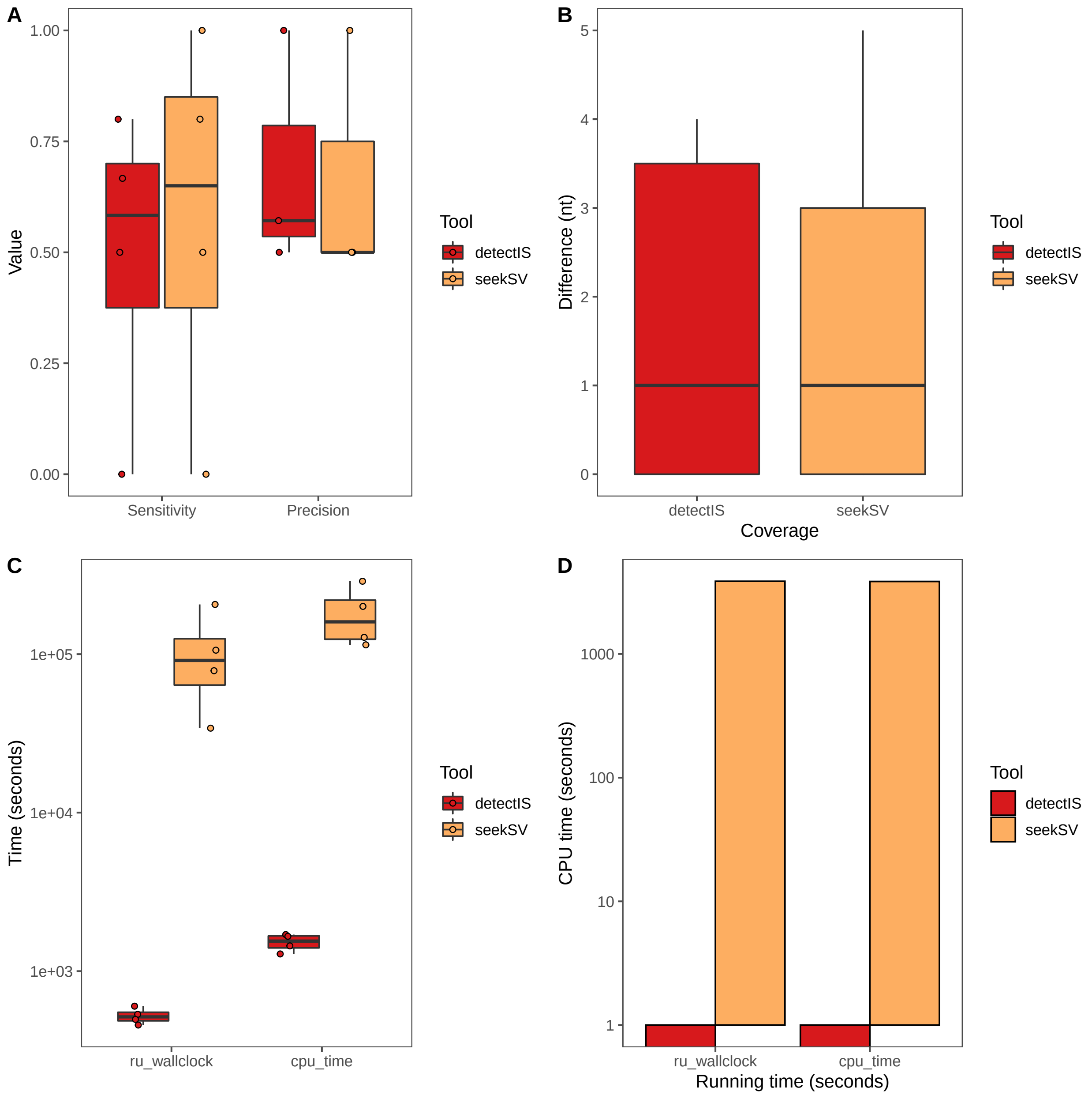

Figure S16 : Benchmark results of detectIS and seekSV on publicly available RNA-seq experiments of four Hepatitis B virus (HBV) positive Hepatocellular carcinoma cell lines with verified chimeric viral-human transcripts (Table S1). A)Box plot reporting precision and sensitivity of the two tools; B)Box plot reporting the absolute values of the differences in nucleotides of the true positives results respect to the real coordinates; C)Box plot reporting wall clock and CPU times of the analyses executed with the two tools; D)Wall clock and CPU times of the index generation step.

Tool	Sample	True positives	False positives	False negatives
detectIS	HKCI-4	4	3	1
seekSV	HKCI-4	4	4	1
detectIS	HKCI-5B	1	0	1
seekSV	HKCI-5B	1	0	1
detectIS	HKCI-7	2	2	1
seekSV	HKCI-7	3	3	0
detectIS	HKCI-11	0	0	1
seekSV	HKCI-11	0	0	1

Table S5 : Benchmark results of detectIS and seekSV on publicly available RNA-seq experiments of four Hepatitis B virus (HBV) positive Hepatocellular carcinoma cell lines with verified chimeric viral-human transcripts (Table S4). In the table are reported true positives, false positives and false negatives for all the experiments analysed using the two tools.
